# Pharmaceutical Impact of *Houttuynia Cordata* and Metformin Combination on High-Fat-Diet-Induced Metabolic Disorders: Link to Intestinal Microbiota and Metabolic Endotoxemia

**DOI:** 10.3389/fendo.2018.00620

**Published:** 2018-10-24

**Authors:** Jing-Hua Wang, Shambhunath Bose, Na Rae Shin, Young-Won Chin, Young Hee Choi, Hojun Kim

**Affiliations:** ^1^Department of Oriental Rehabilitation Medicine, Dongguk University, Goyang, South Korea; ^2^NosQuest, Seongnam-si, South Korea; ^3^College of Pharmacy, Dongguk University-Seoul, Goyang, South Korea; ^4^College of Pharmacy and Integrated Research Institute for Drug Development, Dongguk University-Seoul, Seoul, South Korea

**Keywords:** *Houttuynia cordata*, type 2 diabetes, high fat diet, gut microbiota, endotoxin

## Abstract

**Purpose:** Metformin and *Houttuynia cordata* are representative anti-diabetic therapeutic agents in western and oriental medicinal fields, respectively. The present study examined the therapeutic effects of *houttuynia cordata* extract (HCE) and metformin in combination in a dysmetabolic mouse model.

**Methods:** Metabolic disorders were induced in C57BL/6J mice by high fat diet (HFD) for 14 weeks.

**Results:** Combination of metformin and HCE significantly lowered body weight, abdominal fat, perirenal fat, liver and kidney weights, but did not change epididymal fat in HFD-fed animals. Metformin + HCE treatment markedly attenuated the elevated serum levels of TG, TC, AST, ALT, and endotoxin and restored the depleted HDL level. Both HCE and metformin + HCE treatment ameliorated glucose tolerance and high level of fasting blood glucose in association with AMPK activation. Moreover, treatment with HCE + metformin dramatically suppressed inflammation in HFD-fed animals via inhibition of proinflammatory cytokines (MCP-1 and IL-6) and LPS receptor (TLR4). Histopathological findings showed that exposure of HFD-treated animals to metformin + HCE ameliorated fatty liver, shrinkage of intestinal villi and adipocytes enlargement. Furthermore, HCE and metformin + HCE treatments markedly modulated the abundance of gut Gram-negative bacteria, including *Escherichia coli* and *Bacteriodetes fragilis*, but not universal Gram-positive bacteria.

**Conclusions:** Overall, HCE and metformin cooperatively exert their therapeutic effects via modulation of gut microbiota, especially reduction of Gram-negative bacteria, resulting in alleviation of endotoxemia.

## Introduction

Metabolism is an essential biochemical event in the body that keeps one alive and healthy. However, morbidity due to metabolic diseases such as obesity and diabetes have been continuously increasing and epidemics of these conditions are occurring in both developed and developing countries (1). Many factors, main including genetic and environmental conditions, can disrupt the normal physiological homeostasis, resulting in metabolic disorders (2). Excessive consumption of high-fat-diet (HFD) is one of the main factors that leads to metabolic disorders (3); however, energy imbalance and hereditary reasons do not completely account for the current epidemic status. Recently, increasing studies have reported that the genetic background determines the predisposition of metabolic disorders (4). Metabolic disorders are widely viewed as chronic systemic diseases because they sustain low-grade inflammation due to gut microbial dysbiosis (5). Therefore, intestinal commensal microbiota become another vital factor during the development of metabolic disorders, especially obesity and type 2 diabetes. HFD-altered gut microbiota obviously improve obesity and inflammation via the toll-like receptor 4 signaling pathway (6). In addition, HFD increases intestinal permeability, which leads to elevated serum lipopolysaccharide (LPS) levels because of gut microbiota dysbiosis (5).

*Houttuynia cordata* (HC) is a medicinal and edible herb with an aromatic smell that has long been used in Asia to treat pneumonia, hypertension, constipation, and hyperglycemia via detoxification, reduction of heat and diuretic action. There is accumulating evidence of multiple pharmaceutical effects of HC, such as anti-cancer (7), anaphylactic inhibitory (8), anti-mutagenic (9), anti-inflammatory (10), anti-allergic (11), anti-oxidative (12), anti-viral (13), anti-bacterial (14), anti-obesity (15), and anti-diabetic (16) activities. Moreover, metformin, a well-known biguanide antidiabetic agent that has been used for more than 60 years, exerts multiple-properties such as inhibition of hepatic gluconeogenesis, enhancement of insulin sensitivity and augmentation of peripheral glucose uptake (17, 18). Despite its beneficial impacts, metformin produces a large number of side effects, such as diarrhea, nausea, cramps, vomiting, bloating, lactic acidosis, and abdominal pain, which usually occur in clinics (19). The best-known mechanism of action of metformin is regulation of AMP-activated protein kinase (AMPK) and its downstream signaling pathway (20). Metformin has also been found to reduce hepatic gluconeogenesis and hyperglycemia independently of the AMPK pathway (21). Moreover, metformin induced augmentation of *Akkermansia muciniphila* was shown to improve glucose homeostasis in a HFD induced obese model (22). Although both HC and metformin have beneficial impacts on metabolic disorders, their combination has not been evaluated to date. Therefore, we examined an innovative agent that was formulated by combining HC with metformin to synergistically enhance the therapeutic efficacy and/or decrease side effects relative to HC or metformin alone. Specifically, the therapeutic effects of *Houttuynia cordata* extract (HCE) and metformin in combination were investigated using high-fat-diet (HFD) induced metabolic dysfunction of mice model. We also explored the corresponding potential mechanisms, especially regarding alteration of gut microbiota and systemic endotoxemia.

## Materials and methods

### Houttuynia cordata extract (HCE) and metformin

*Houttuynia cordata* was obtained from the pharmacy of Dongguk University Ilsan International Hospital (Goyang, South Korea). After grinding, powder of *Houttuynia cordata* was extracted by 5 L ethanol recycling reflux for 4 h. The extract was then filtered and vacuum lyophilized at −70°C, which gave a 5.82% yield. The HCE contained 3.63% quercitrin, 0.45% quercetin and 0.99% of isoquercitrin (23). Metformin was purchased from Sigma-Aldrich (St. Louis, MO, USA).

### Animals and experimental schedule

**Figure 1:**
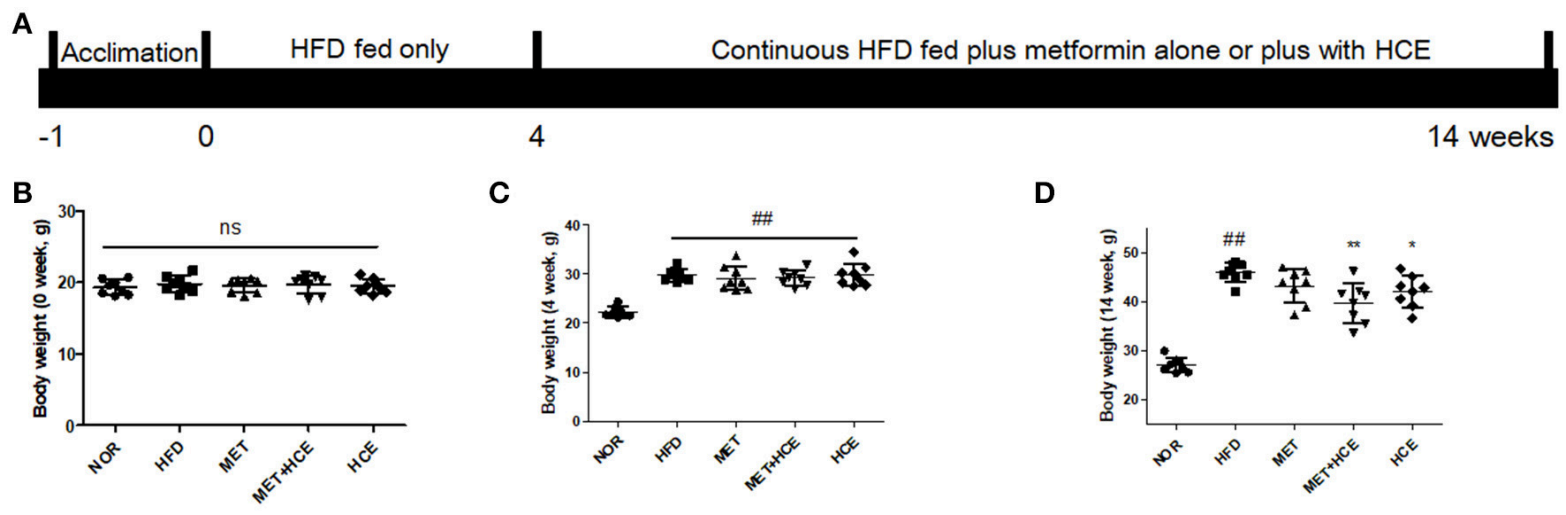
Animal experimental schedule and body mass. The experimental design is illustrated intuitively **(A)** and body weight of mice were recorded at the start **(B)**, week 4 **(C)** and week 14 of the experiment **(D)**. Data were expressed as the means ± SD and evaluated using one-way ANOVA followed by the LSD *post-hoc* test. ^##^*P* < 0.01 compared to the normal group; ^*^P < 0.05 compared to the HFD group; ^**^P < 0.01 compared to the HFD group (*n* = 7). “ns” means none statistic significant.

The animal study was approved by the Institutional Animal Care and Use Committee (IACUC-2015-037) of Dongguk University and conducted in accordance with the Guide for the Care and Use of Laboratory Animals (Institute of Laboratory Animal Resources, Commission on Life Sciences, National Research Council, USA; National Academy Press: Washington D.C., 1996). Specific-pathogen-free (SPF) C57BL/6j male mice were obtained from Koatech (Gyeonggi-do, South Korea). After 1 week of acclimatization, 40 mice were equally divided into five groups by average body weight. The normal group was fed a control diet (Table S1) (AIN-93G diet) for 14 weeks, while the other four groups were continuously fed 60% calorie high fat diet (HFD) (Table S1) for 14 weeks (Figure 1A). From week five to 14, among the HFD-fed mice, eight were treated with metformin (100 mg/kg/day; metformin group), eight with HCE (400 mg/kg/day), eight were treated with a combination of metformin (50 mg/kg/day) and HCE (200 mg/kg/day) and the remaining eight were administrated distilled water as a negative control group. The experimental doses of metformin and HCE were determined based on their clinical dosages and the Guidance for Industry (2005). On the last experimental day, fresh stool samples were collected, and after 12 h of fasting all the animals were weighed and anesthetized using Zoletil (tiletamine-zolazepam, Virbac, Carros, France) and Rompun (xylazine-hydrochloride, Bayer, Leverkusen, Germany) in a 1:1 v/v combination. Blood was then collected from the ventral aorta and rapidly transferred into a BD Vacutainer (Franklin Lakes, NJ, USA) for serum separation. Liver, intestine and fat tissues were removed, weighed and rapidly stored in liquid nitrogen for future analysis.

### Oral glucose tolerance test (OGTT)

In the last week of the animal experiment, rats were fasted for 12 h, and then orally dosed with glucose solution (2 g/kg, Sigma-Aldrich, St. Louis, MO, USA). The blood glucose levels were then measured by ACCU-CHEK Active (Mannheim, Germany) using blood collected from the tail vain at 0, 30, 60, 90, 120 min post-glucose dosing. The OGTT results were also expressed as areas under the curves (AUC) to evaluate the degree of glucose tolerance impairment.

### Serum biochemical analysis

Blood collected from the ventral aorta was centrifuged at 3,000 × g for 15 min to separate the serum. The serum levels of triglyceride (TG), total cholesterol (TC), high density lipoprotein (HDL), aspartate transaminase (AST), and alanine transaminase (ALT) were subsequently determined using commercial enzymatic assay kits (Asan Pharmaceutical Co., Seoul, Korea) according to the manufacturer's instructions.

### Serum endotoxin analysis

Serum endotoxin levels was measured using a Limulus Amebocyte Lysate (LAL) kit (ENDOSAFE, SC, USA) according to the kit manufacturer's instructions. Briefly, 10× dilutions of mice serum samples were added to the kit supplied plate and wells were spiked with 5 EU/mL standard. Following the addition of 100 μL of LAL reagent, the kinetic absorbance of the mixture was measured at 405 nm and the reaction onset times of the samples were compared to the standard curve.

### Oil red O and H&E staining

Liver, jejunum and adipose tissues were embedded in FSC 22 frozen section compound (Leica Biosystems, Richmond, IL, USA), then frozen and sectioned at 5 mm using a Leica CM1860 Cryostat (Leica Microsystems, Nussloch, Germany). Sections were then stained with oil red O solution or hematoxylin and eosin (Cayman chemical, USA), after which they were mounted on silicone-coated slides (Leica, USA) and examined using an Olympus BX61 microscope (Tokyo, Japan) and photographed using an Olympus DP70 digital camera (Tokyo, Japan).

### Real-time PCR for analyzing gene expression in liver tissue

Total RNA was isolated from liver tissues using TRIsure™ (BIOLINE, MA, USA). cDNA was synthesized using an AccuPower RT premix kit (Bioneer, Daejeon, Korea) and real-time PCR amplification reactions were conducted with the corresponding primers (Table S2) using a LightCycler® FastStart DNA Master SYBR Green kit and a LightCycler instrument (Roche Applied Science, Indianapolis, ID, USA). The reaction was conducted in a total reaction volume of 20 μl consisting of PCR mix, 1 μl of cDNA, and gene-specific primers (10 pmol each). The relative gene expression was represented by 2^−ΔCt^ using β-actin as a housekeeping gene for normalization, where Ct is the crossing threshold value and ΔCt = Ct (target gene) - Ct (β-actin).

### Western blot analysis

Mice liver tissues were homogenized in RIPA buffer (Abcam, USA) containing protease and phosphatase inhibitors (Abcam, USA). The supernatant was isolated, and total protein concentrations was measured using a BCA kit (Thermo Scientific, USA). Denatured proteins were separated in 10% SDS-PAGE gel, then transferred to polyvinylidene fluoride (PVDF) membrane (GE Healthcare Life Science, Germany) using the Mini-PROTEAN Tetra Cell System (BioRad Laboratories Inc., CA, USA). The membranes were blocked by 5% skim milk with TBST and Tris-buffered saline, then washed with Tween 20 for 1 h and treated with primary antibody (1:10,000) overnight at 4°C. Samples were subsequently incubated with horseradish peroxidase-conjugated secondary antibodies (1:2,000, beta actin manufactured by Santa Cruz, USA; AMPK, phosphorylated-AMPK and GLUT2 manufactured by Cell Signaling, USA) for 1 h. Detailed information regarding the antibodies is shown in Table S3. Finally, the band on membranes were detected using SUPEX ECL solution and photographed using a FUJIFILM LAS3000 Image Analyzer (FUJI, Japan).

### Fecal microbial analysis using RFLP (restriction fragment length polymorphism) and real-time PCR

Fecal genomic DNA was isolated using a QIAamp DNA Stool Mini Kit (Qiagen, CA, USA) for RFLP and real-time PCR analyses. The 16S rRNA genes were PCR amplified using the universal bacterial primers 27F (5′-AGAGTTTGATCCTGGCTCAG-3′), which were 5′ end-labeled with 5-FAM and 1492R (5′-GGTTACCTTGTTACGACTT-3′). PCR amplification was conducted using an initial denaturation step at 94°C for 3 min, followed by 30 cycles of 1 min at 94°C, 45 s at 53°C and 2 min at 72°C. The reaction was completed with a final primer elongation step at 72°C for 10 min. Following confirmation by agarose gel electrophoresis, PCR products were digested with the MspI restriction enzyme (TaKaRa, Shiga, Japan). The DNA samples containing the extension products were then added to Hi-Di formamide (Applied Biosystems) and GeneScan™ 1200 LIZ® Size Standard (Applied Biosystems, Foster City, CA, USA). The mixture was subsequently incubated at 95°C for 5 min, placed on ice for 5 min, then analyzed using a 3730XL DNA analyzer (Applied Biosystems, Foster City, CA, USA). Next, T-RFLP electropherograms were imaged using GeneMapper® v5.0 and the Peak Scanner 2 software (Applied Biosystems). The relative peak areas of each terminal restriction fragment (TRF) were determined by dividing the area of the peak of interest by the total area of peaks within the following threshold values: lower threshold = 50 bp; upper threshold = 500 bp. Data were normalized by applying a threshold value for relative abundance at 0.5% and only TRFs with higher relative abundances were included in the remaining analyses.

Roche LightCycler FastStart DNA Master SYBR Green was used to conduct real-time PCR using the LightCycler 480 system (Roche Applied Science, Indianapolis, IN, USA). The primer sequences targeting the 16S rRNA gene of the bacteria are listed in Table S2. The standard conditions for the PCR amplification reactions were applied as previously described (23). The relative quantification of bacterial abundance is shown by 2^−Ct^ calculations (Ct, threshold cycle). The final results are expressed as normalized fold values relative to the normal group.

### Cells culture and viability assay

All cell lines were cultured in an incubator at 37°C in presence of humidified air of 5% CO_2_. Mouse myoblasts (C2C12; Korea Cell Line Bank, Seoul, Korea) were cultured in DMEM or RPMI-1640 (GIBCO, Carlsbad, CA, USA) supplemented with 10% fetal bovine serum (FBS, GIBCO, CA, USA), 2 mM L-glutamine (GIBCO, Carlsbad, CA, USA), 100 U/ml penicillin (GIBCO, Carlsbad, CA, USA), and 100 μg/ml streptomycin (GIBCO, Carlsbad, CA, USA). The cell viability was determined using an EZ-cytox enhanced cell viability assay kit (DOGEN, Seoul, Korea). Briefly, after achieving approximately 80% confluency, the cells were treated for 24 h with quercitrin or quercetin (Sigma, USA) at 1, 5, 10, 20, 50, or 100 μM concentrations. EZ-Cytox was added to the cells 2 h prior to the end of the treatment schedule. Following completion of the reaction, the culture media were transferred to a fresh 96-well microplate. The absorbance of the wells was then read at 450 nm (650 nm as a reference wavelength) (Spectramax Plus, Molecular Devices, CA, USA). The viability of the control cells, in terms of their absorbance, was set to 100%.

### Determination of glucose uptake *in vitro*

The C2C12 cells were seeded at 1 × 10^4^ cells per well in 96-well black, clear bottom culture plates (Greiner Bio-One, Frickenhausen, Germany) together with 10% FBS/DMEM (GIBCO, Carlsbad, CA, USA) plus antibiotics (GIBCO, Carlsbad, CA, USA) for 24 h at 37°C in presence of humidified air of 5% CO_2_. The cells were then incubated in glucose-free DMEM supplemented with 2% horse serum for 96 h until more than 90% differentiation was achieved (approximately 96 h). Next, cells were treated with 10 mM glucosamine (Sigma-Aldrich, MO, USA) and/or 200 nM insulin (Sigma, USA) for 4 h. Finally, cells were treated with metformin (750 μM) alone or in combination with HCE (100 μg/mL), quercitrin (8 μM) or quercetin (2 μM) for 12 h and subsequently treated with 75 μg/mL of 2-deoxy-2-[(7-nitro-2,1,3-benzoxadiazol-4-yl)amino]-D-glucose (2-NBDG, Life Technologies, CA, USA) for 2 h. Eventually, the uptake of 2-NBDG by the cells was measured by fluorescence microscopy (Olympus BX-61, Tokyo, Japan) and determined using a SpectraMax M3 fluorescence reader (Molecular Devices, CA, USA) with excitation and emission wavelengths of 475 and 515 nm, respectively.

### Statistical analysis

All experimental data were analyzed by one-way ANOVA followed by the LSD (least significant difference) *post-hoc* test using SPSS 17.0 (Chicago, IL, USA). The results were expressed as the means ± standard deviations (SD) and a *P* < 0.05 was considered statistically significant.

## Results

### Reduction of body, organ, and fat weights

Following termination of the experimental schedule at week 14, the body, fat, liver, and kidney weights of HFD-fed mice were significantly higher compared to animals fed normal diet treatment, as expected. Treatment of HFD-fed animals with both HCE and metformin + HCE markedly reduced the body, fat, liver and kidney weights. Moreover, exposure of HFD-fed mice to metformin reduced the abdominal fat weight, but less significantly than the HCE and metformin + HCE treatments (Figures 1B–D, Table 1). Furthermore, metformin treatment did not produce any significant effect on body, perirenal, epididymal or total fat of HFD-fed animals. Although not statistically significant, combination of metformin and HCE showed greater anti-obesity effects than either compound alone (Table 1).

**Table 1:** Comparison of body, fat and organ weights.

**Groups**	**normal**	**HFD**	**metformin**	**metformin + HCE**	**HCE**
Body weight gain (g/week)	0.48 ± 0.07	1.62 ± 0.22^##^	1.40 ± 0.23	1.04 ± 0.46^**^	1.22 ± 0.37^*^
Food intake (g/week)	16.9 ± 0.8	17.4 ± 0.8	15.6 ± 0.8^**^	16.5 ± 0.8^*^	16.4 ± 0.7^*^
Food efficiency ratio	0.028	0.094	0.085	0.059	0.072
Abdominal fat (g)	0.29 ± 0.05	1.51 ± 0.09^##^	1.23 ± 0.31^*^	0.74 ± 0.36^**^	1.12 ± 0.23^**^
Perirenal fat (g)	0.29 ± 0.11	0.99 ± 0.09^##^	0.98 ± 0.14	0.80 ± 0.15^*^	0.83 ± 0.14^*^
Epididymal fat (g)	0.86 ± 0.21	1.93 ± 0.29	2.31 ± 0.46	2.20 ± 0.38	2.20 ± 0.43
Total fat (g)	1.49 ± 0.37	4.40 ± 0.38^#^	4.52 ± 0.47	3.76 ± 0.80^*^	4.14 ± 0.53
Liver weight (g)	0.93 ± 0.08	1.86 ± 0.15^##^	1.40 ± 0.28^**^	1.16 ± 0.33^**^	1.24 ± 0.21^**^
Kidney weight (g)	0.32 ± 0.02	0.39 ± 0.03^##^	0.34 ± 0.03^**^	0.36 ± 0.02^*^	0.34 ± 0.03^**^

TC, total cholesterol; HDL, high density lipoprotein; TG, triglyceride. Data were expressed as Mean ± SD, different letters indicate significantly different at measured by One-Way ANOVA followed by LSD (P < 0.05, n = 8).

# P < 0.05,

## P < 0.01 compared to the normal group;

* P < 0.05,

** *P < 0.01 compared to the HFD group*.

### Amelioration of serum lipid parameters and hepatic transaminases

As expected, treatment with HFD significantly increased the levels of serum TG, TC, AST and ALT, and markedly decreased the levels of serum HDL. Combined metformin and HCE treatment significantly attenuated the levels of TG, TC, AST and ALT, and significantly increased the levels of serum HDL in the HFD group. Metformin treatment alone only significantly lowered the level of serum ALT, while HCE treatment alone markedly lowered the levels of serum TC and ALT relative to the HFD group. Overall, combination of metformin and HCE group ameliorated the serum lipid profile and liver transaminases to a greater extent than metformin or HCE alone (Table 2).

**Table 2:** Comparison of serum biochemistry parameters.

**Groups**	**normal**	**HFD**	**metformin**	**metformin + HCE**	**HCE**
TG (mg/dL)	159 ± 38	182 ± 28	195 ± 26	154 ± 18^*^	176 ± 32
TC (mg/dL)	128 ± 15	187 ± 24^##^	177 ± 11	155 ± 21^**^	173 ± 33^*^
HDL (mg/dL)	27.19 ± 4.72	18.67 ± 2.29^##^	20.42 ± 1.45	22.95 ± 2.04^**^	17.66 ± 1.99
AST	24.88 ± 8.62	35.87 ± 6.29^#^	32.24 ± 7.96	22.87 ± 4.81^**^	28.49 ± 7.42
ALT	6.43 ± 3.15	23.73 ± 4.90^##^	14.56 ± 9.19^*^	6.39 ± 3.97^**^	9.82 ± 5.78^**^

TC, total cholesterol; HDL, high density lipoprotein; TG, triglyceride. Data were expressed as Mean ± SD, different letters indicate significantly different at measured by One-Way ANOVA followed by LSD (P < 0.05, n = 7).

# P < 0.05,

## P < 0.01 compared to the normal group;

* P < 0.05,

** P < 0.01 compared to the HFD group

### Improvement of hyperglycemia and glucose tolerance *in vivo* and glucose uptake *in vitro*

**Figure 2:**
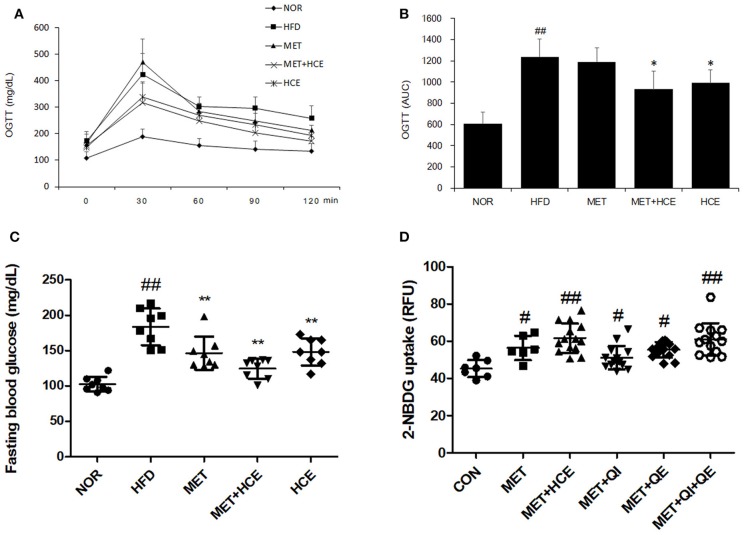
Glucose related parameters *in vivo* and *in vitro*. Impact of metformin either alone or in combination with HCE on insulin sensitivity and glucose tolerance in OLETF rats. Oral glucose tolerance tests (OGTTs) **(A)** of the animals were conducted in the last week and the areas under the curves (AUCs) **(B)** were constructed as described in the Materials and Methods section. Fasting blood glucose **(C)** was also recorded on the last day of the *in vivo* experiment. The glucose uptake ability **(D)** of metformin (750 μM), HCE (100 μg/mL) and its main compounds (8 μM of quercitrin, 2 μM of quercetin) were assessed in C2C12 cells. Data were expressed as the means ± SD and evaluated using one-way ANOVA followed by the LSD *post-hoc* test. **(B)** ^##^*P* < 0.01 compared to the normal group; ^*^P < 0.05 compared to the HFD group (*n* = 5). **(C)** ^##^*P* < 0.01 compared to the normal group; ^**^P < 0.01 compared to the HFD group (*n* = 7). **(D)** ^#^*P* < 0.05 compared to the control group; ^##^*P* < 0.01 compared to the control group (*n* = 14).

As anticipated, HFD treatment significantly increased the fasting glucose relative to the normal group. Both metformin and HCE alone and in combination notably lowered the high fasting blood glucose (FBG) relative to HFD treatment. Combined treatment with metformin and HCE showed more efficient reduction of hyperglycemia than treatment with metformin and HCE alone. In addition, OGTT (AUC) was markedly increased by HFD treatment relative to the normal group, while HCE and HCE + metformin treatment significantly reduced the OGTT (AUC) relative to HFD treatment. Finally, HCE + metformin treatment showed more effective amelioration of glucose tolerance than HCE alone (Figures 2A–C, Table S4).

The *in vitro* results showed that treatment of C2C12 cells with either metformin alone or metformin + HCE remarkably elevated the glucose uptake. Interestingly, metformin in combination with quercitrin plus quercetin treatment, but not metformin + quercitrin or quercetin, exhibited a similar ability for glucose uptake in HepG2 cells as metformin + HCE treatment (Figure 2D).

### Alleviation of systemic endotoxin

Serum endotoxin level was significantly elevated in the HFD group relative to the normal group. However, the metformin + HCE group more significantly reduced the serum endotoxin concentration than HCE or metformin alone relative to the HFD group.

### Histopathological alteration

**Figure 3:**
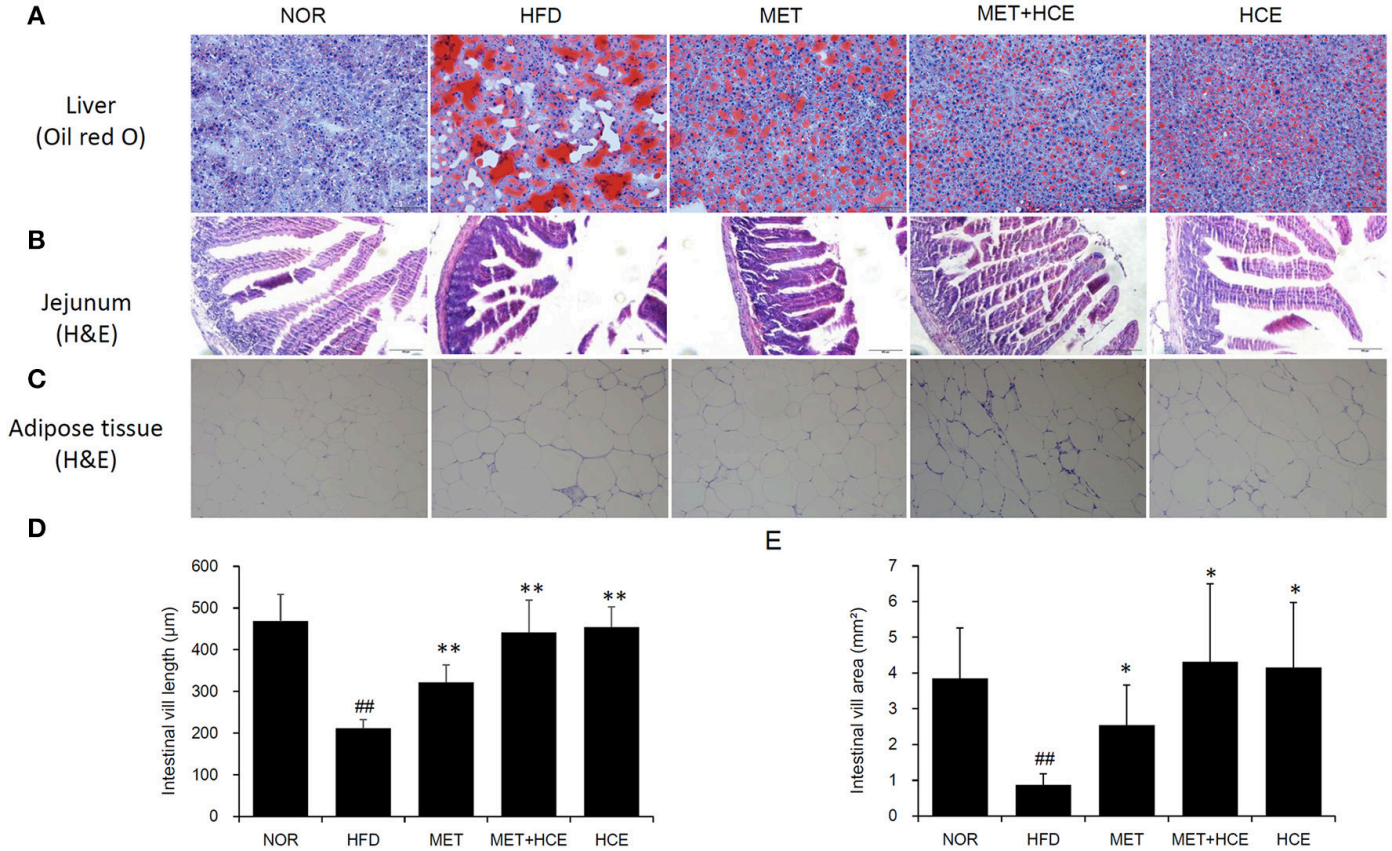
Histopathological analysis. On the final experimental day, the liver **(A)**, jejunum **(B)** and adipose tissue **(C)** were removed rapidly, after which tissue sections were prepared and stained with oil red O or hematoxylin and eosin. Histological examination of the tissue sections was conducted under a light microscope (200 × magnification). Calculated length **(D)** and volume **(E)** of the intestinal villi are shown. Data were expressed as the means ± SD and evaluated using one-way ANOVA followed by the LSD *post-hoc* test. ^##^*P* < 0.01 compared to the normal group; ^*^P < 0.05 compared to the HFD group; ^**^P < 0.01 compared to the HFD group (*n* = 3).

Staining of hepatic tissue with oil red o revealed that HFD treatment induced lipid droplet deposition in the liver (Figure 3A). Additionally, HFD treatment markedly decreased the length and volume of intestinal villi and obviously reduced the size of adipocytes relative to the normal group (Figures 3B–E). However, these alterations were recovered in all of the medicine-treated groups. Indeed, the hepatic lipid accumulation, intestinal villi atrophy, and adipocytes enlargement in the HFD-fed animals were more prominently ameliorated by metformin + HCE treatment than metformin or HCE treatment alone.

### Activation of AMPK and GLUT2

**Figure 4:**
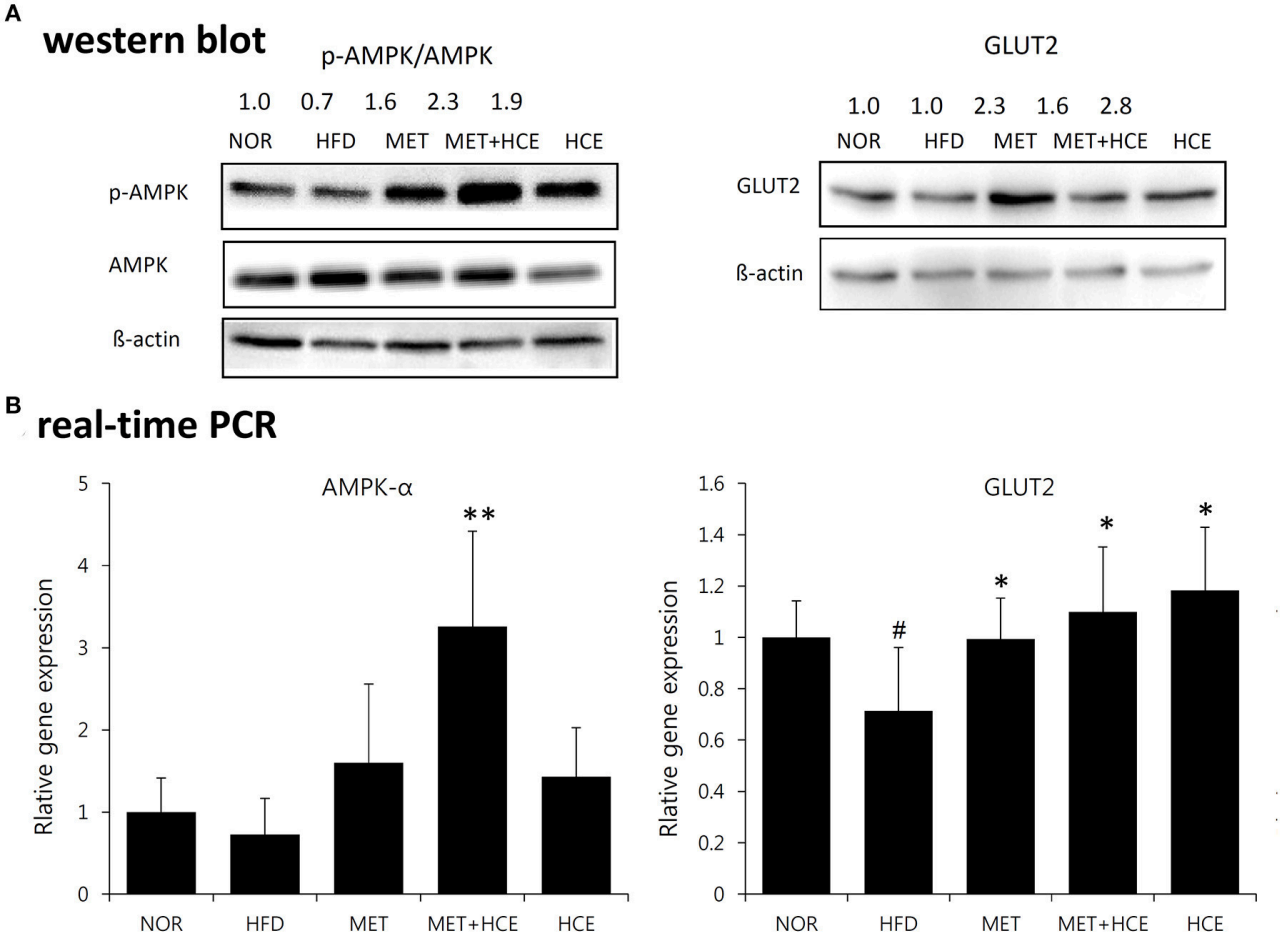
Activation of AMPK and GLUT2. Impact of metformin and HCE alone or combination on the activation of hepatic AMPK and GLUT2 as valued by Western blotting **(A)** and real-time PCR **(B)**. ^#^*P* < 0.05 compared to the normal group; ^*^P < 0.05 compared to the HFD group; ^**^P < 0.01 compared to the HFD group (*n* = 7).

Treatment of HFD-fed animals with metformin + HCE, but not metformin or HCE alone, resulted in a significant increase in hepatic gene expression of AMPK. Moreover, treatment of HFD-fed animals with metformin + HCE enhanced the pAMPK/AMPK ratio. However, treatment of HFD-fed animals with metformin or HCE led to less enhancement of the pAMPK/AMPK ratio than their combination. Moreover, exposure of HFD-fed animals to all treatments significantly elevated hepatic gene expression of GLUT2 and markedly increased the hepatic GLUT2 protein level (Figure 4).

### Attenuation of inflammation

**Figure 5:**
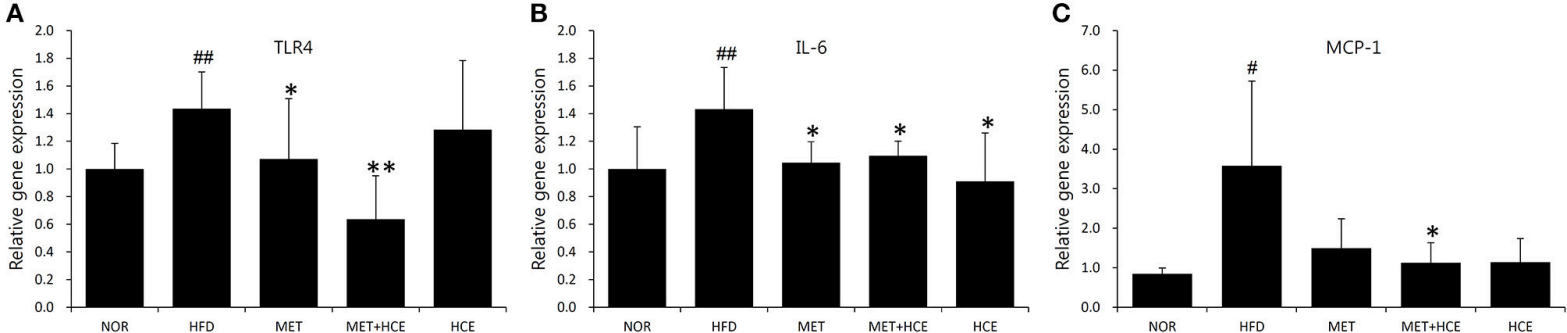
Suppression of inflammatory cytokines. The gene expression of the TLR4 **(A)**, IL-6 **(B)** and MCP-1 **(C)** were analyzed using real-time PCR in liver tissue. Data were expressed as the means ± SD and statistically evaluated using one-way ANOVA followed by the LSD *post-hoc* test. ^#^*P* < 0.05 compared to the normal group; ^##^*P* < 0.01 compared to the normal group; ^*^P < 0.05 compared to the HFD group; ^**^P < 0.01 compared to the HFD group (*n* = 7).

AS expected, HFD treatment significantly up-regulated gene expression of the TLR4 and downstream signaling proteins, such as IL-6 and MCP-1, relative to the normal group. Nevertheless, HCE + metformin treatment showed greater inhibition of the TLR4 and MCP-1 expression than HFD treatment rather relative to metformin or HCE alone (Figure 5).

### Modification of gut microbial distribution

**Figure 6:**
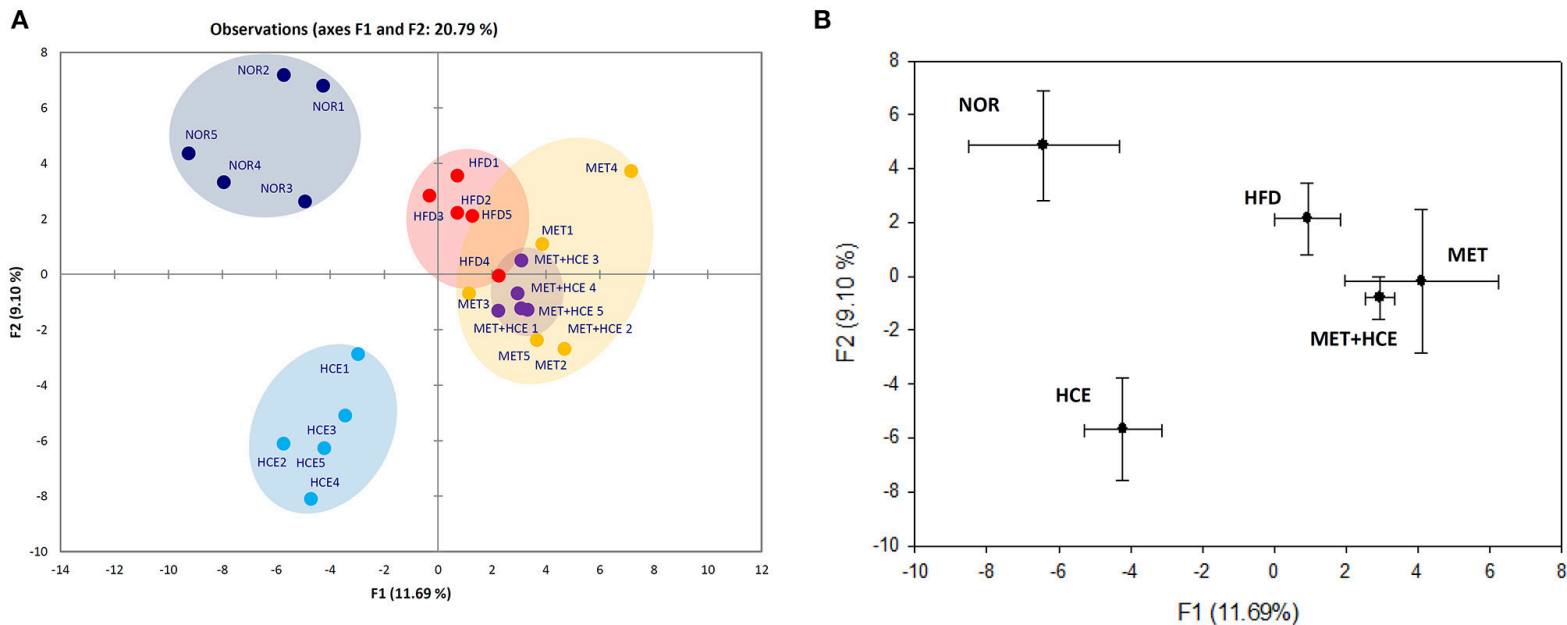
RFLP and PCoA analysis of mice feces. **(A)** Before sacrifice, the mice fecal samples were collected and the microbial communities were analyzed by restriction fragment length polymorphism (RFLP) as described in the Materials and Methods section. **(B)** PCoA analysis of the RFLP data was conducted and diagramed using XLSTAT to further evaluate the similarities between bacterial communities.

**Figure 7:**
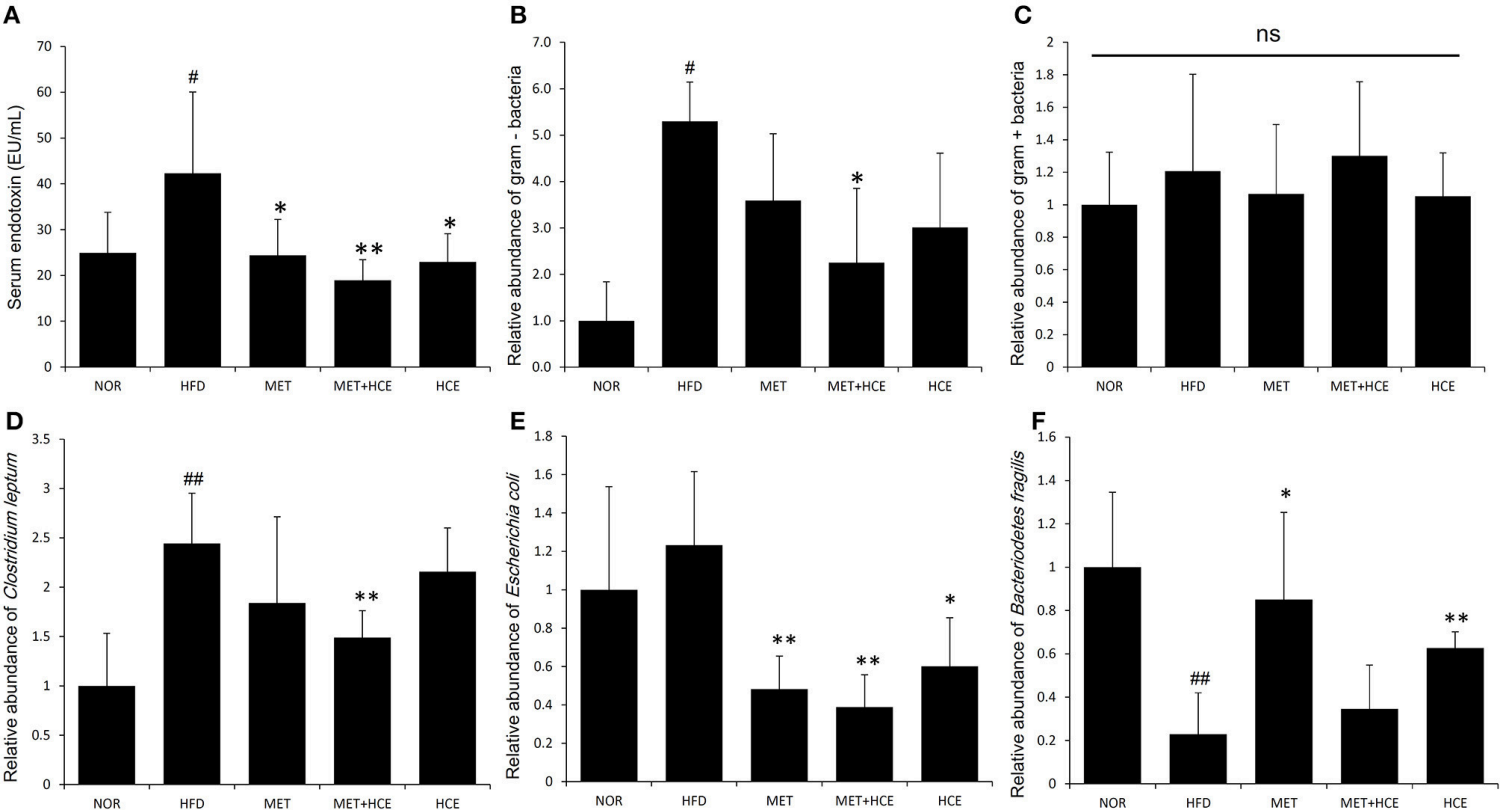
Quantitative determination of systemic endotoxins and relative abundance of gut microbiota. On the final experimental day, blood was collected from the animals and the serum endotoxin level **(A)** was determined as described in the Materials and methods. Stool samples were collected and the abundance of the 16S rRNA gene of the bacterial strains **(B–F)** was determined as described in the Materials and Methods section. The results are expressed as normalized fold values relative to the normal group. Data were expressed as the means ± SD and evaluated using one-way ANOVA followed by the LSD *post-hoc* test. ^#^*P* < 0.05 compared to the normal group; ^##^*P* < 0.01 compared to the normal group; ^*^P < 0.05 compared to the HFD group; ^**^P < 0.01 compared to the HFD group (*n* = 7). “ns” means none statistic significant.

PCoA analysis of RFLP data revealed unique characteristics of the gut microbial community in normal, HFD, metformin and HCE groups. More specifically, the distribution pattern of the gut microbial community in the metformin + HCE group had more similarity with the metformin alone group than with other groups (Figure 6). Exposure to HFD resulted in a significant increase in the abundance of Gram-negative bacteria in the animals. Additionally, treatment with HFD-fed animals with metformin + HCE, but neither metformin nor HCE alone, significantly decreased the population of universal Gram-negative bacteria. Conversely, exposure of HFD-fed animals to all three medicines significantly reduced the population of *Escherichia coli*. No significant differences in the abundance of universal Gram-positive bacteria were observed among groups. However, exposure of HFD-fed animals to metformin + HCE, but neither metformin nor HCE alone, significantly decreased the population of *Clostridium leptum*. In contrast, treatment of HFD-fed animals with metformin or HCE alone resulted in a greater increase in *Bacteriodetes fragilis* abundance when compared to HFD-fed animals treated with metformin + HCE (Figure 7).

## Discussion

Although substantial studies have shown that HC and metformin individually could improve metabolic activities (24, 25), to the best of our knowledge, this is the first report to evaluate the impact of combined treatment with metformin and HCE in a dysmetabolic animal model induced by HFD. More specifically, the major goal of this study was to examine whether the edible formulation of the medicinal herb HC can exert certain synergic effects on the activity of metformin or relieve the side effects of this antidiabetic drug, as well as to elucidate the underlying mechanism of any overserved effects. Based on the actual clinical dosage calculated by a conversion formula from FDA guidance (26), we selected 100 mg/kg of metformin, 400 mg/kg of HCE and half of this dose of metformin (50 mg/kg) together with half the dose of HCE (200 mg/kg) for this investigation. As a representative anti-hyperglycemia agent, metformin significantly ameliorated the FBG in HFD-treated animals. Similarly, HCE treatment significantly reduced the FBG level in HFD-fed animals; however, combination of the metformin and HCE more effectively lowered the FBG than metformin or HCE alone at their higher doses. OGTT, the most widely used procedure for evaluating whole body glucose tolerance, has often been employed to assess insulin sensitivity (27, 28). Indeed, since last 20 years, various indices of insulin sensitivity/resistance using the data from OGTT are documented (29). In the present study, treatment of HFD-fed animals with metformin and HCE in combination led to a greater improvement in OGTT parameters than higher doses of metformin or HCE alone, suggesting the synergistic beneficial impact of these two therapeutic agents on glucose tolerance as well as insulin sensitivity/resistance. Furthermore, in a previous study, using relevant *in vitro* and *in vivo* models, we showed that treatment with metformin + HCE was more beneficial than metformin alone in the improvement of glucose uptake, insulin secretion, glucose metabolism and insulin sensitivity (23).

Our results revealed that the level of quercetin and quercitrin in its glycoside form in HCE were 0.363 and 0.045 mg/g, respectively. These two compounds are active pharmaceutical ingredients of HCE known to have potential antioxidant and anti-inflammatory activities (30). Quercetin shares a common mechanism with metformin in elevating glucose uptake, which is mediated via AMPK activation and upregulation of GLUT expression (31). Our results indicated that HCE assisted metformin in further phosphorylation and gene expression of AMPK. Exposure of HFD-fed animals to all treatments significantly elevated glucose uptake ability via an increase in gene expression of GLUT2 as well as the hepatic level of transporter protein. Thus, our *in vitro* and *in vivo* findings indicate that the combination of metformin and HCE may ameliorate hyperglycemia and glucose tolerance via cooperative augmentation of glucose uptake. It is worth noting that HCE boosts these effects, which is likely because of the collaborative action of quercetin and quercitrin rather than other components.

As expected, obesity, fatty liver, and fatty kidney pathophysiological states were induced in animals in response to long-term HFD feeding as supported by a noteworthy increase in body, fat, liver and kidney weights. In parallel, histopathological evidence, such as marked hepatic lipid accumulation and increased adipocyte population in the adipose tissue of HFD-fed animals also indicated that HFD generates grievous lipid dysmetabolism. As in previous studies (23), treatment with either HCE or metformin ameliorated the symptoms of obesity and fatty liver in the present investigation. Meanwhile, HFD destroyed the morphology of intestinal villus; however, these effects were obviously ameliorated by HCE and/or metformin treatment. Interestingly, treatment of HFD-fed animals with HCE and metformin in combination at their half doses was found to be more effective at reducing the body weight, liver weight and fat weight, especially the weight of abdominal and perinephric fats, than treatment with HCE or metformin alone at their original doses. Notably, none of the aforementioned treatments altered the epididymal fat content of HFD-fed animals.

As circulating lipid markers, the levels of serum TG, TC, and HDL indicate the status of holistic lipid metabolism. Chronic consumption of HFD induces dyslipidemia and the development of fatty liver (32). Previous reports demonstrated that treatment of HFD-fed rats with metformin or HC alone depleted the increased serum levels of TG and TC, and that this was accompanied with increased serum HDL levels (33, 34). Interestingly, in the present study, HCE + metformin treatment more effectively restored the dysregulated lipid metabolism than HCE or metformin alone in HFD-fed animals. Additionally, as expected, the serum levels of both hepatic transaminases AST and ALT, the sensitive indicators of various liver injuries including fatty liver, were found to be significantly higher in the HFD group than the normal group, which was in keeping with the aberrated histological architecture of the liver in the former group. Overall, our results revealed that treatment of HFD-fed animals with HCE + metformin was more effective than treatment with either compound alone at restoring liver morphology and reducing the serum levels of AST and ALT.

Lipopolysaccharides (LPS), which are also known as endotoxins, exists in the outer membrane of Gram-negative bacteria, where they trigger entotoxemia (5). Metabolic endotoxemia-induced chronic low-grade inflammation has been deemed a vital hallmark of metabolic diseases such as obesity and type 2 diabetes (35). Previous reports have shown that both metformin and HC possess anti-inflammatory activities (36, 37). Furthermore, metformin prevents a number of diseases that are associated with endotoxin insult of Gram-negative bacteria (38–40). In our study, combination of metformin and HCE more significantly attenuated the level of endotoxin in the circulatory system of HFD-fed animals than either compound alone. This is further supported by our findings regarding the significant reduction in abundance of fecal universal Gram-negative bacteria without any modulation in the population of fecal universal Gram-positive bacteria in HFD-fed mice in response to treatment with metformin + HCE, but not with metformin or HCE alone. The significant suppression of gene expression of both proinflammatory cytokine IL-6 and inflammatory chemokine MCP-1, as well as the potent inhibition of TLR4 in HFD-fed mice by metformin + HCE also indicates a feasible mechanism for the cooperative effects of this combination on the anti-inflammatory action against endotoxemia.

For the last few years, the relationship between various diseases and gut commensal microbiota has been widely investigated worldwide (41). Gut microbial composition, which can be altered by HFD (42), plays a vital role in the development of metabolic diseases through regulation of host energy homeostasis and redundancy in fat accumulation (43). Therefore, gut microbial modulation is regarded as a feasible strategy for ameliorating metabolic diseases. Indeed, previous studies have revealed that both metformin and medicinal herbs can ameliorate obesity and related endotoxemia, probably via alteration of the distribution of gut microbiota (22, 44). According to our RFLP analysis, exposure of HFD-fed animals to metformin + HCE caused a more pronounced modulation of the gut microbial population than other treatments. The more similar profile of gut microbiota between the metformin + HCE group and the metformin alone group indicates that metformin potentially restrained the HCE-induced gut microbiota shift. Interestingly, similar to dietary fiber (45), combination of metformin and HCE notably improved glycemia and reduced *Clostridium leptum* in HFD-induced obese animals. Therefore, it is conceivable that HCE together with metformin may exert prebiotic effects leading to significant reduction in the population of gut Gram-negative bacteria, including *Escherichia coli*.

Taken together, our findings suggest that HCE assists metformin in the improvement of obesity, glucose tolerance, hyperglycemia, and hyperlipidemia. This is more likely mediated by reduction of endotoxin and inflammatory stress through regulation of the gut microbial community, particularly *Clostridium leptum* and Gram-negative bacteria including *Escherichia coli*. Thus, it is conceivable that combined treatment with *Houttuynia cordata* and metformin may provide a more efficient strategy for the treatment of patients with metabolic syndrome, particularly T2D and hyperlipidemia. The gut microbiota responsible for contributing the synergistic effects of *Houttuynia cordata* on metformin need to be further explored in future studies.

## Author contributions

J-HW wrote manuscript. SB edited and rewrote some parts of manuscript. NS analyzed microbiota data. Y-WC and YC involved in study design and data analysis. HK conceived and designed the study.

### Conflict of interest statement

The authors declare that the research was conducted in the absence of any commercial or financial relationships that could be construed as a potential conflict of interest.
