# Supplementary material for: Pharmaceutical Impact of Houttuynia Cordata and Metformin Combination on High-Fat-Diet-Induced Metabolic Disorders: Link to Intestinal Microbiota and Metabolic Endotoxemia
Source: Front Endocrinol (Lausanne). 2018 Oct 24;9:620. doi: 10.3389/fendo.2018.00620 (PMC6208002; doi:10.3389/fendo.2018.00620)
Supplement: Supplementary file 1 [file Table_1.DOCX]

| Table S1 Animal diets formulation and ingredients | | | | |
| --- | --- | --- | --- | --- |
| Formulation | Normal diet (AIN-93G) | | High Fat Diet | |
|  | gm% | kcal% | gm% | kcal% |
| Protein | 20 | 20 | 27 | 20 |
| Carbohydrate | 64 | 64 | 25 | 20 |
| Fat | 7 | 16 | 36 | 60 |
| Kcal/kg | 4,000 |  | 5,333 |  |
|  | | | | |
| Ingredient | g | kcal | g | kcal |
| Casein (from milk) | 200 | 800 | 200 | 800 |
| Corn starch | 397,486 | 1,590 | 47,536 | 190 |
| Sucrose | 100 | 400 | 0 | 0 |
| Dextrose | 132 | 528 | 132 | 528 |
| Cellulose | 50 | 0 | 50 | 0 |
| Soybean oil | 70 | 630 | 25 | 225 |
| Lard | 0 | 0 | 245 | 2,205 |
| Mineral mixture | 35 | 0 | 35 | 0 |
| Vitamin mixture | 10 | 40 | 10 | 40 |
| TBHQ | 0.014 | 0 | 0.014 | 0 |
| L-Cystine | 3 | 12 | 3 | 12 |
| Choline bitartrate | 2.5 | 0 | 2.5 | 0 |
| Total | 1,000 | 4,000 | 750.1 | 4,000 |
| Abbreviations: TBHQ, tertiary butylhydroquinone | | | | |

| Table S2. Primer sequences used for real-time PCR | | | |
| --- | --- | --- | --- |
| Bacteria | Primer sequence | OAT |  |
| Gram negative bacterium | 5'-AYG ACG TCA AGT CMT CAT GG-3' | 65°C |  |
|  | 5'-AAC TGG AGG AAG GTG GGG AY-3' |  |  |
| Gram positive bacterium | 5'-AGGAGGTGATCCAACCGC-3' | 60°C |  |
|  | 5'-GAYGACGTCAARTCMTCATGC-3' |  |  |
| *Echerichia coli* | 5'-GAC CCG GCA CAA GCA TAA GC-3' | 65°C |  |
|  | 5'-CCA CCT GCA GCA ACA AGA GG-3' |  |  |
| Clostridium leptum | 5'-GCACAAGCAGTGGAGT-3' | 50°C |  |
|  | 5'-CTTCCTCCGTTTTGTCAA-3' |  |  |
| Bacteriodetes fragilis | 5'-GAGAGGAAGGTCCCCCAC-3' | 60°C |  |
|  | 5'-CGCTACTTGGCTGGTTCAG-3' |  |  |
| *Faecalibacterium prausnitzii* | 5'-GGA GGA AGA AGG TCT TCG G-3' | 60°C |  |
|  | 5'-AAT TCC GCC TAC CTC TGC ACT-3' |  |  |
| b-actin | 5'-GCAAGTGCTTCTAGGCGGAC-3' | 54°C |  |
|  | 5'-AAGAAAGGGTGTAAAACGCAGC-3' |  |  |
| *TRL-4* | 5'-CCTGATGACATTCCTTCTTCAAC-3' | 57°C |  |
|  | 3'-TTGTTTCAATTTCACACCTGGATAAA-3' |  |  |
| *IL-6* | 5′-AGTTGCCTTCTTGGGACTGA-3′ | 55°C |  |
|  | 5′-CAGAATTGCCATTGCACAAC-3′ |  |  |
| *MCP-1* | 5′-GCATCCACGTGTTGGCTCA-3′ | 57°C |  |
|  | 5′-CTCCAGCCTACTCATTGGGATCA-3′ |  |  |
| Abbreviations: OAT, optimized annealing temperature | | | |

| Table S3. Antibodies for western blot analysis | | | | |
| --- | --- | --- | --- | --- |
| Peptide/protein target | Manufacturer, catalog #, and/or name of individual providing the antibody | Species raised in; monoclonal or polyclonal | Dilution used |  |
| Beta-actin | Santa Cruz, #sc-47778 | Mouse monoclonal | 1:2000 |  |
| AMPK | Cell Signaling, #2532 | Rabbit polyclonal | 1:1000 |  |
| Phosphorylated AMPK | Cell Signaling, #2535 | Rabbit polyclonal | 1:1000 |  |
| GLUT-2 | Abcam, #95256 | Rabbit polyclonal | 1:1000 |  |

Table S4. Raw data of oral glucose tolerance test and Area Under the Curve

| 0 min (mg/dL) | NOR | HFD | MET | MET+HCE | HCE |
| --- | --- | --- | --- | --- | --- |
| 1 | 138 | 163 | 102 | 194 | 153 |
| 2 | 75 | 129 | 199 | 142 | 182 |
| 3 | 107 | 153 | 176 | 147 | 128 |
| 4 | 114 | 200 | 176 | 117 | 142 |
| 5 | 108 | 219 | 157 | 180 | 137 |
| Mean | 108.4 | 172.8 | 162.0 | 156.0 | 148.4 |
| SD | 22.5 | 36.3 | 36.7 | 30.9 | 20.8 |
|  |  |  |  |  |  |
| 30 min (mg/dL) | NOR | HFD | MET | MET+HCE | HCE |
| 1 | 216 | 416 | 378 | 261 | 387 |
| 2 | 159 | 375 | 422 | 282 | 346 |
| 3 | 170 | 331 | 581 | 365 | 287 |
| 4 | 186 | 531 | 543 | 285 | 364 |
| 5 | 219 | 467 | 425 | 388 | 313 |
| Mean | 190.0 | 424.0 | 469.8 | 316.2 | 339.4 |
| SD | 26.9 | 78.1 | 87.2 | 56.4 | 48.6 |
|  |  |  |  |  |  |
| 60 min (mg/dL) | NOR | HFD | MET | MET+HCE | HCE |
| 1 | 190 | 248 | 277 | 197 | 247 |
| 2 | 120 | 288 | 250 | 263 | 341 |
| 3 | 150 | 325 | 269 | 257 | 213 |
| 4 | 143 | 331 | 301 | 221 | 252 |
| 5 | 172 | 321 | 326 | 309 | 293 |
| Mean | 155 | 302.6 | 284.6 | 249.4 | 269.2 |
| SD | 26.96294 | 34.78937 | 29.50085 | 42.8579 | 49.16503 |
|  |  |  |  |  |  |
| 90 min (mg/dL) | NOR | HFD | MET | MET+HCE | HCE |
| 1 | 188 | 241 | 216 | 173 | 213 |
| 2 | 115 | 263 | 247 | 166 | 306 |
| 3 | 122 | 312 | 252 | 205 | 197 |
| 4 | 125 | 345 | 294 | 161 | 226 |
| 5 | 160 | 322 | 231 | 306 | 224 |
| Mean | 142 | 296.6 | 248 | 202.2 | 233.2 |
| SD | 31.0564 | 43.14279 | 29.35132 | 60.50372 | 42.29303 |
|  |  |  |  |  |  |
| 120 min (mg/dL) | NOR | HFD | MET | MET+HCE | HCE |
| 1 | 174 | 217 | 179 | 160 | 207 |
| 2 | 110 | 240 | 217 | 138 | 172 |
| 3 | 120 | 245 | 219 | 191 | 181 |
| 4 | 115 | 341 | 231 | 147 | 204 |
| 5 | 154 | 247 | 219 | 231 | 205 |
| Mean | 134.6 | 258 | 213 | 173.4 | 193.8 |
| SD | 27.97856 | 47.91659 | 19.79899 | 37.93811 | 16.14621 |
|  |  |  |  |  |  |
| Mean (mg/dL) | NOR | HFD | MET | MET+HCE | HCE |
| 0 min | 108.4 | 172.8 | 162 | 156 | 148.4 |
| 30 min | 190 | 424 | 469.8 | 316.2 | 339.4 |
| 60 min | 155 | 302.6 | 284.6 | 249.4 | 269.2 |
| 90 min | 142 | 296.6 | 248 | 202.2 | 233.2 |
| 120 min | 134.6 | 258 | 213 | 173.4 | 193.8 |
|  |  |  |  |  |  |
| SD | NOR | HFD | MET | MET+HCE | HCE |
| 0 min | 22.5 | 36.3 | 36.7 | 20.8 | 30.9 |
| 30 min | 26.9 | 78.1 | 87.2 | 75.4 | 56.4 |
| 60 min | 27.0 | 34.8 | 29.5 | 49.2 | 42.9 |
| 90 min | 31.1 | 43.1 | 29.4 | 42.3 | 60.5 |
| 120 min | 28.0 | 47.9 | 19.8 | 16.1 | 37.9 |
|  |  |  |  |  |  |
| AUC | NOR | HFD | MET | MET+HCE | HCE |
| 1 | 750 | 1095 | 1011.5 | 808 | 1027 |
| 2 | 486.5 | 1110.5 | 1127 | 851 | 1170 |
| 3 | 555.5 | 1167 | 1299.5 | 996 | 851.5 |
| 4 | 568.5 | 1477.5 | 1341.5 | 799 | 915 |
| 5 | 682 | 1343 | 1170 | 1208.5 | 1001 |
| Mean | 608.5 | 1238.6 | 1189.9 | 932.5 | 992.9 |
| SD | 105.7 | 165.9 | 133.4 | 173.3 | 121.1 |
